# Fungicides disrupt total and endophytic phyllosphere bacterial communities but a salicylic acid hyperimmune mutant shows microbiome resilience

**DOI:** 10.1093/ismeco/ycag102

**Published:** 2026-04-14

**Authors:** Stacey A Vincent, Paul F Devlin

**Affiliations:** Department of Biological Sciences, Royal Holloway University of London, Egham, Surrey TW20 0EX, United Kingdom; Department of Biological Sciences, Royal Holloway University of London, Egham, Surrey TW20 0EX, United Kingdom

**Keywords:** plant, phyllosphere, fungicide, Arabidopsis, salicylic acid, immune response, microbiome, metabarcoding, diversity, bacteria

## Abstract

Stable colonization of plants by beneficial microbes enhances disease resistance, nutrient uptake, and stress tolerance. Disruption of these communities often reduces plant fitness. The phyllosphere microbiome is especially vulnerable to agrochemicals. In this study we examined how synthetic fungicides affect the phyllosphere bacterial community of *Arabidopsis thaliana*. Application of several widely-used fungicides led to a pronounced decrease in bacterial diversity and depletion of beneficial taxa in both surface and internal leaf microbial communities. Moreover, these microbial responses were influenced by the host plant’s genetic background. We previously showed that the phyllosphere microbiomes of plants exhibiting heightened salicylic acid-driven immune responses are enriched in xenobiotic degradation traits. We, therefore, examined whether the disrupted phyllosphere of one such line, the *fhy3 far1* mutant, is buffered against fungicide-induced dysbiosis. The *fhy3 far1* mutant showed reduced fungicide-induced microbiome disruption in both surface and endophytic microbiomes across both systemic and contact fungicides, supporting the hypothesis that innate plant immunity may help buffer against collateral damage from chemical treatments. Our identification of fungicide-resilient microbial taxa holds promise for the development of next-generation biostimulant products and, additionally, our findings raise the possibility that salicylic acid-mediated immunity could be strategically leveraged as a complementary tool alongside traditional fungicides.

## Introduction

The plant microbiome comprises an intricate and dynamic community of microorganisms that significantly contribute to plant health and productivity. These microbial populations influence various aspects of plant biology, including growth, development, immunity, and environmental resilience [1–4]. Microorganisms inhabit almost every part of the plant, from roots and stems to leaves and flowers, and form specialized communities adapted to each niche [5]. This microbiome is taxonomically and functionally diverse, consisting of bacteria, fungi, archaea, viruses, and a variety of other microorganisms [6]. A single plant can harbor thousands of microbial species, many of which engage in complex interactions with the host plant and with each other [7].

Among the different plant compartments, the rhizosphere, the soil region surrounding plant roots, has been studied the most extensively. This zone is a hub of microbial activity and plays a pivotal role in shaping plant-microbe relationships. Rhizospheric microbes frequently form mutualistic partnerships with plants, facilitating nutrient acquisition, improving resistance to pathogens, and enhancing tolerance to various environmental stresses [8, 9]. In contrast, the above-ground parts of the plant, collectively known as the phyllosphere, also support a diverse microbial community, although typically at lower abundances than the rhizosphere. Despite this lower microbial density, phyllosphere residents can have considerable effects on plant physiology, particularly by promoting resistance to diseases and abiotic stresses [6, 10].

An imbalance in these microbial communities, a state known as dysbiosis, can predispose plants to infections and negatively impact growth and yield [11]. Microorganisms within both the rhizosphere and phyllosphere can exist as either epiphytes—living on the plant surface—or as endophytes, which colonize internal tissues. Endophytic microbes are increasingly recognized for their beneficial roles, offering support similar to surface microbes but often interacting more intimately with plant cells due to their location in the apoplast, the intercellular space within plant tissues [1, 3, 12, 13].

Plants are not passive hosts in these interactions. They influence microbiome composition through the release of biochemical signals, such as root exudates, which can attract or repel specific microbial taxa. The role of root exudates in shaping the rhizosphere microbiome has been well documented [14–17]. The phyllosphere, however, presents a more challenging environment for microbial colonization. Exposed to extreme temperatures, ultraviolet radiation, and limited moisture and nutrients, the phyllosphere lacks the microbial richness of the soil, making colonization more stochastic and susceptible to environmental fluctuations [10, 18]. Nonetheless, several studies have identified a core microbiome in the phyllosphere, indicating that plants may selectively maintain beneficial microbes to preserve essential functions [6, 11, 19–22]. This is supported by a recent large scale modelling study of human gut microbiomes which provides evidence that the core microbiome may represent a beneficial community selected through coevolution for its beneficial traits in maintaining overall stability and functionality [23].

Efforts to manipulate the plant microbiome for agricultural benefit—referred to as microbiome engineering—have gained significant momentum. One promising application is the use of microbial consortia as biopesticides to mitigate the devastating impacts of pathogens and pests on crop yields. Global agricultural losses due to biotic stressors can range between 17% and 30% in major crop systems [24]. Traditional methods for managing plant disease, including the widespread use of synthetic pesticides, are effective but problematic. While pesticides help reduce crop losses, they also pose serious environmental concerns, such as contaminating ecosystems and harming non-target organisms [25, 26]. Additionally, excessive and continuous pesticide application has led to the evolution of resistant pests and pathogens, compromising long-term efficacy [27].

In light of these challenges, researchers are exploring more sustainable alternatives. The phyllosphere has emerged as a particularly attractive target for microbiome-based solutions, given its role as a common entry point for foliar pathogens and its harboring of naturally antagonistic microbes with antipathogenic capabilities [28–30]. Despite this potential, attempts to engineer stable and effective synthetic microbial communities (SynComs) for the phyllosphere or rhizosphere have generally not translated well to field conditions, largely because these introduced communities struggle to compete with or integrate into pre-existing microbiomes [31].

This failure underscores the need to deepen our understanding of plant-microbe dynamics, particularly the traits that promote persistence and functional integration of SynComs. A completely pesticide-free agricultural system is unlikely, but incorporating microbiome engineering into integrated pest management (IPM) strategies could substantially reduce chemical inputs. An ideal SynCom would be not only effective against pathogens but also resilient to chemical pesticides, ensuring functionality even under typical agricultural conditions.

Unfortunately, research on the effect of pesticides on plant microbiomes has mostly focused on the soil, where negative consequences on symbiotic functions are well established [32, 33]. Given the phyllosphere’s greater exposure to environmental factors and direct contact with foliar sprays, it is expected to be even more vulnerable to chemical disturbance [34]. Supporting this, recent work in *Nicotiana tabacum* demonstrated that fungicide application altered the structure of the beneficial core bacterial community in the phyllosphere [35]. However, other work has shown negligible impacts on phyllosphere bacteria. Noel *et al.* [36] who assessed the impact of fungicides on the phyllosphere microbiome of maize and soybean, found no evidence of any global alteration composition of phyllosphere prokaryote communities suggesting that bacterial impacts vary based on fungicide type. However, they did observe effects on non-target fungi, which indirectly influenced the co-occurrence networks between fungi and bacteria. Indeed, there are several hypotheses that may explain off-target effects of fungicides on bacterial communities. Off target toxicity may mean that fungicides directly negatively impact bacteria either due to shared common metabolic targets or due to additional toxic impacts specific to bacteria [37]. Alternatively, as fungi and bacteria often compete for the same resources or exist in a delicate balance, removal of fungi may result in ecosystem disruption, reducing network complexity, which can indirectly hinder associated bacterial populations [38]. Equally, some fungicides may provide a carbon source, promoting certain bacterial taxa capable of metabolizing the fungicide [39].

To assess how fungicide exposure alters taxonomic composition and potential functional traits of the phyllosphere microbiome, the present study evaluated the impact of a diverse array of commonly used fungicides on the phyllosphere bacterial communities of *Arabidopsis thaliana*. Active ingredients were selected based on their differing modes of action and regulatory status within the European Union. These were applied using a standardized surfactant without commercial additives, to isolate the effects of the fungicides themselves. Additionally, drawing from previous work showing that salicylic acid (SA)-driven immune responses enrich the phyllosphere microbiome for traits associated with xenobiotic degradation and tolerance of oxidative stress, the study examined whether one of the mutants examined in that study was buffered against fungicide-induced dysbiosis. To this end, experiments were also conducted using the *fhy3 far1* mutant, which exhibits constitutive SA signalling.

In wild-type plants, all fungicides tested led to a reduction in total phyllosphere microbial diversity and altered microbial composition, indicating community disturbance and potential dysbiosis. However, these negative effects were significantly mitigated in the *fhy3 far1* mutant, suggesting a possible protective role of SA-mediated hyperimmunity. When examining the endophytic bacterial communities specifically, only the systemic fungicide Azoxystrobin significantly reduced diversity in wild-type plants, with minimal effect observed in the mutant. This highlights how systemic and contact fungicides may differently influence endophytic microbiota due to their differing interactions with plant tissues.

These results support the hypothesis that SA-induced immunity may select for microbial communities with inherent resilience to environmental disturbances, including chemical stressors. Identifying such resilient taxa and understanding their functional traits could inform the development of robust, protective SynComs for future microbiome engineering efforts.

## Materials and methods

### Plant materials and growth conditions

*Arabidopsis thaliana* No-0 ecotype and *fhy3–4 far1–*2 lines were described previously [21]. Seeds were sown on damp compost mixture consisting of John Innes No.3 soil, Levington M3 soil and perlite (6:6:1 vol/vol). Plants were germinated and grown in a temperature-controlled growth room on a long day (LD) 16/8 h light/dark cycle at 20°C at a Photon Flux Density of 127 μmol m^−2^ s^−1^. All seeds were previously bulked and collected from plants grown in LD conditions as described here, across multiple generations to control for the initial inoculum. Three repeat experiments were carried out to form three completely independent biological replicates.

### Fungicide application

Plants were sprayed with either 0.25 g/l active fungicidal ingredient dissolved in ethanol and sterile H_2_O (1:5 v/v) and Tween-20 (0.05% v/v) or with solvent alone 14 and 28 days after sowing (DAS), resulting in a g/l dose comparable to the individual maximum dose typical in agricultural practice. All fungicides were supplied by Sigma–Aldrich, Gillingham, UK. A listing of the active fungicidal ingredients and mode of action used in study is provided in Table 1.

**Table 1:** Active fungicidal ingredients and mode of action used in study. Modified from Baibakova *et al.* [40] with the addition of aminopyrimidine information [41, 42].

Active ingredient	Class	Mode of action
Prochloraz	Benzimidazoles	Inhibition of ergosterol biosynthesis
Azoxystrobin	Strobilurins	Cytochrome electron transport inhibition
Fludioxonil	Phenylpyrroles	Disruption of cellular respiration, cell membrane functionality and inhibition of growth
Cyprodinil	Aminopyrimidines	Inhibition of methionine biosynthesis and hydrolytic enzyme secretion
Difenoconazole	Triazoles	Sterol biosynthesis inhibition
Dimethomorph	Morpholines	Inhibition of mycelium formation and ergosterol synthesis

### DNA extraction

For each biological replicate, aerial plant tissues from three plants were combined to a total of up to 100 mg. These tissues were harvested using sterile forceps, flash-frozen in liquid nitrogen and stored at −80°C. Where only endophytic microbial communities were examined, microorganisms that inhabit the leaf surface were washed from tissues prior to freezing according to an adaptation of a previously described method [11]. In detail, leaves were soaked in 5% bleach (v/v) for 1 min before soaking in 75% ethanol for 1 min. Both methods have been shown to be extremely effective at removing epiphytic bacteria [43, 44]. The leaf tissue was then rinsed mechanically 2–3 times in sterile H_2_O before being dried out on blotting paper. The leaves were then flash-frozen and stored at −80°C as described above. For each biological replicate, frozen aerial plant tissue from three individual plants of each genotype 35 DAS were harvested using sterile forceps, flash-frozen in liquid nitrogen and stored at −80°C. Frozen plant tissue was homogenized by adding glass silica beads at a sufficient quantity to fully cover the lysis buffer added downstream; flash-frozen again and vortexed using the Scientific Industries™ Vortex-Genie™ 2 at maximum rpm for 30 s. The samples were immediately flash frozen a third time and the lysis buffer from the Qiagen® DNeasy® Plant Mini Kit was added. The samples were vortexed for a further 3.5 min, or until the tissue was completely homogenized. The remaining steps of the DNA extraction were carried out according to the manufacturer’s instructions and DNA was eluted in nuclease-free water preheated to 55°C.

### Polymerase chain reaction amplification

A universal 16S rRNA primer pair, 799F (5′-AAC MGG ATT AGA TAC CCK G-3′) and 1193R (5′-ACG TCA TCC CCA CCT TCC-3′), targeting the V5-V7 16S rRNA regions was used to amplify extracted DNA and generate sequences for NGS metabarcoding. This region was chosen over the commonly-used V4 region for our study as it shows reduced amplification of chloroplast 16s rRNA sequences, allowing greater sequencing depth among bacteria [45–47]. All polymerase chain reactions (PCRs) were carried out in triplicate 25 μl reactions. The PCR was carried out in reactions each containing 1X GoTaq® Hot Start Green Master Mix; 0.1 μM 799F primer; 0.1 μM 1193R primer and 2 μl of DNA template. PCR cycling conditions were: an initial denaturation at 94°C for 4 min; touch-down annealing from 63°C–53°C, decreasing in 1°C increments per cycle for 1 min; and extending at 72°C for 1 min. After reaching the final annealing temperature of 53°C this was repeated for 30 cycles with a final extension step at 72°C for 7 min. Technical triplicates were pooled and separated on a 1% low melting temperature agarose gel; and the band corresponding to the expected amplicon size was excised. No template control samples run alongside plant samples to ensure that there was no laboratory contamination.

### Isolation of microbial DNA from plant host DNA

Measures were taken to minimize the abundance of plant host organelle DNA in the 16S rRNA amplicons prepared for NGS from whole leaves. The host mtDNA amplicon that is present following amplification of the V5–V7 16S rRNA region is larger in length than the 16S rRNA amplicon of interest. Therefore, these amplicons were separated by gel electrophoresis; and the lower-length amplicon of interest was excised. The DNA was then purified from the gel using a Qiagen® Gel Extraction kit according to the manufacturer’s instructions. Co-amplification of cpDNA was minimized by the use of the chloroplast-excluding forward primer 799F [45–47].

### Illumina MiSeq next-generation sequencing

16S rRNA amplicons for each sample were then used as input for library preparation. DNA libraries were prepared from amplicons by tagmentation using the Nextera™ DNA Flex Library Prep Kit from Illumina according to the manufacturer’s instructions. Library concentrations were quantified using a Qubit 4 fluorimeter and the library length was assessed using an Agilent Bioanalyzer TapeStation. The library was subsequently sequenced on the Illumina MiSeq platform, generating paired-end reads. The quality of output reads was assessed using FastQC (v.0.72 for Galaxy) [48].

### Bioinformatic analysis

Microbiome analysis was carried out with mothur on the command line (v.1.44.3) using modifications to the mothur standard operating procedure [49]. Firstly, forward and reverse reads were aligned into contigs using the Needleman alignment method and poorly paired reads were discarded. Screening was performed to remove reads that contained ambiguous sequences and homopolymers longer than the reference database later used for classification. Sequences identified as being potentially chimeric were also removed. Bacterial 16S rRNA sequences were then classified using the mothur-formatted SILVA SEED alignment and taxonomy reference files v.138 [50] using the Wang classification algorithm. Undesired taxa, such as cpDNA and mtDNA were subsequently removed, and the remaining sequences clustered into operational taxonomic units (OTUs) at 97% sequence similarity. Alpha and beta diversity analyses were additionally carried out in mothur (v.1.44.3) [49] on data subsampled to the size of the smallest sample in the analysis, using the Chao1 estimator of species richness, and Shannon diversity and Shannon evenness indexes for alpha diversity. For comparisons of Simpson diversity indices, replicates were averaged for each genotype and inter-condition comparisons were made using the Past4 Diversity t-test function [51]. *P*-values were adjusted using the Benjamini–Hochberg procedure in R. Indicator species analysis was performed using the IndVal.g statistic with permutation testing (999 permutations) in the “indicspecies” R package [52]. Bray–Curtis dissimilarities were calculated from Hellinger-transformed OTU tables (square-root of per-sample relative abundances) prior to NMDS ordination in R [53, 54]. Differences in community composition were assessed using permutational multivariate analysis of variance (PERMANOVA; adonis2, vegan) on the same Bray–Curtis dissimilarity matrix, with significance assessed by permutation [55, 56].

## Results

### Fungicide treatment reduces bacterial diversity in the phyllosphere microbiome

To explore the off-target consequences of fungicide application on the total bacterial phyllosphere microbiome, a set of widely-used contact and systemic fungicides was applied to healthy, mature *A. thaliana* WT (No-0) plants at two developmental stages: 14 and 28 DAS. Plants were subsequently harvested at 35 DAS, and the resulting leaf samples underwent 16S rRNA gene metabarcoding to assess microbiome alterations. Across three biological replicates per treatment and genotype, a total of 1 944 715 high-quality, taxonomically classified 16S rRNA sequences were obtained (Supplementary Table 1).

**Figure 1:**
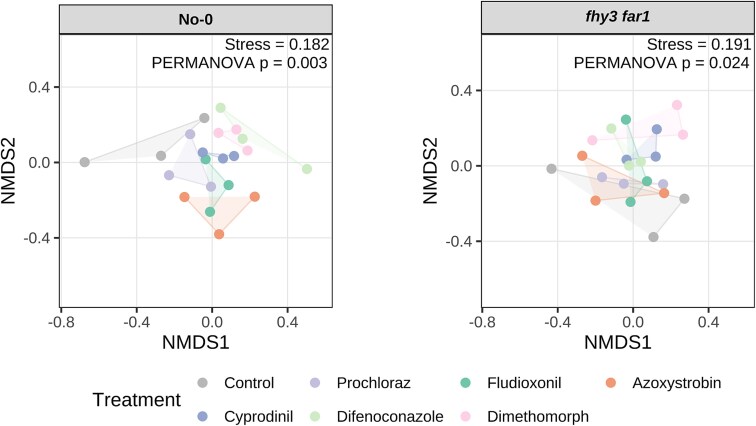
NMDS ordination of fungicide-treated leaf bacterial communities characterized by classification of 16S rRNA sequences. WT and *fhy3 far1 A. thaliana* genotypes, which had been sprayed with fungicide or control at 14 and 28 DAS, were harvested 35 DAS (*n* = 3). All samples were subsampled down to the size of the sample with the lowest number of sequences prior to analysis. Bray–Curtis dissimilarities were calculated from Hellinger-transformed OTU tables (square-root of per-sample relative abundances) prior to NMDS ordination in R [53, 54]. Differences in community composition between treatments within each genotype were assessed using PERMANOVA, with significance assessed by permutation. PERMANOVA *P*-values are reported on the ordination panels [55, 56].

Analysis of OTUs using non-metric multidimensional scaling (NMDS) revealed high reproducibility across replicates and distinct clustering between fungicide-treated and buffer-treated control samples (Figure 1), with PERMANOVA analysis indicating significant fungicide-induced shifts in the phyllosphere microbiota.

**Figure 2:**
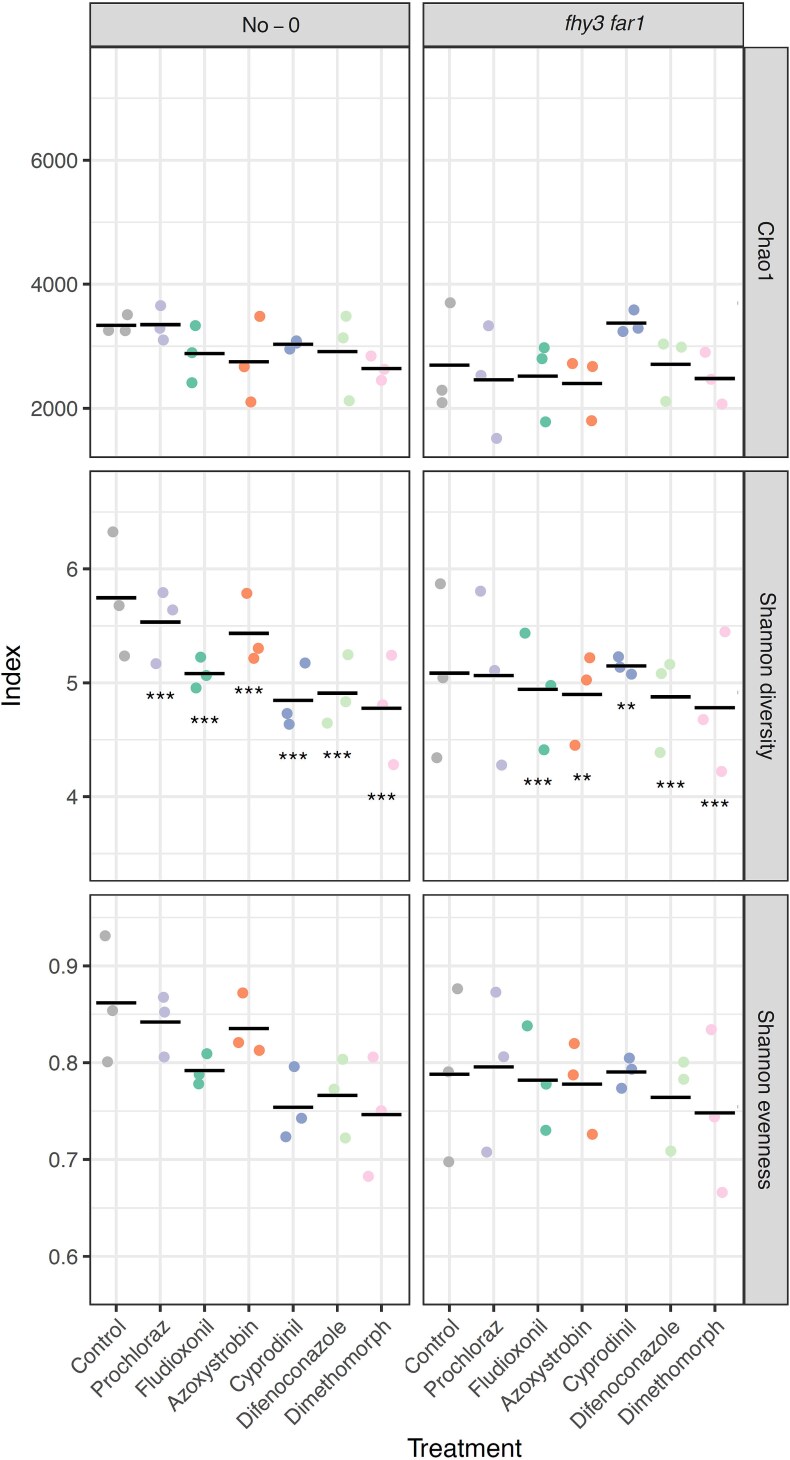
Alpha diversity estimations of the diversity of bacterial phyllospheric communities. WT and *fhy3 far1 A. thaliana* lines were harvested 35 DAS (*n* = 3). The Chao1 estimator shows the estimated species richness; and the Shannon diversity and Shannon evenness index show the community diversity and evenness, respectively. All samples were subsampled down to the size of the sample with the lowest number of sequences before calculating. Alpha diversity metrics were calculated using mothur (v.1.44.3) [49] and inter-condition comparisons were made using the Past4 Diversity t-test function [51]. *P*-values were adjusted using the Benjamini–Hochberg procedure in R for controlling the false discovery rate in multiple hypothesis testing. Asterisks represent statistical significance, where “*” indicates *P* < .05; “**” indicates *P* < .01; and “***” indicates *P* < .001.

Reduced diversity in the leaf microbiome is associated with reduced fitness in the host plant and dysbiosis the phyllosphere [11]. Shannon alpha diversity indices showed that all fungicides caused a statistically significant reduction in bacterial diversity compared to the control group (Figure 2). This trend was most pronounced following application of systemic fungicides, particularly Cyprodinil, Difenoconazole, and Dimethomorph. Consistent patterns were also observed in measures of species richness and evenness (Figure 2).

**Figure 3:**
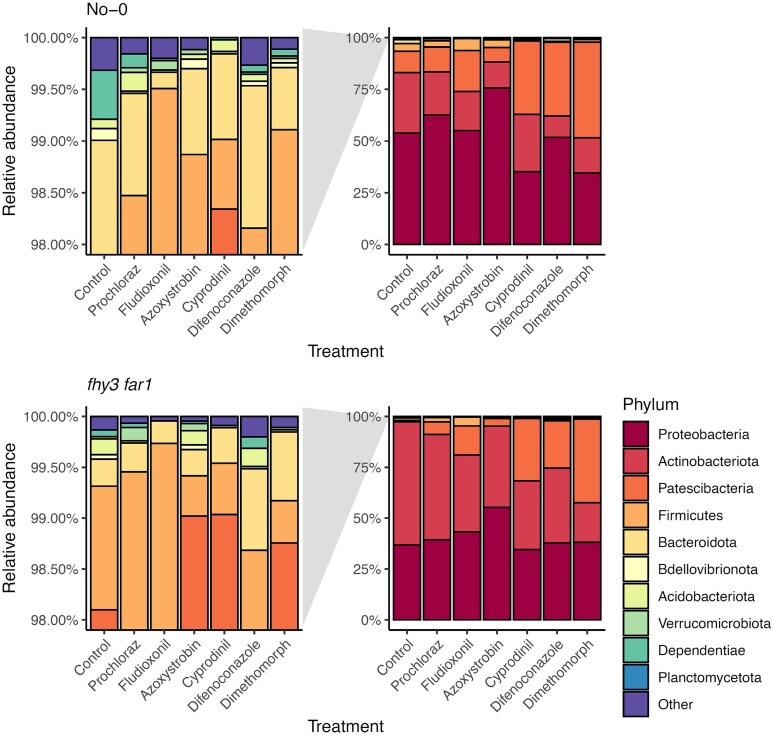
Relative abundances of classified phyllospheric bacteria after treatment with fungicides. Microbial DNA was isolated from aerial tissues of WT and *fhy3 far1 A. thaliana* lines 35 DAS (*n* = 3). Bacteria classified to at least the phylum level were included in the analysis. The panels on the left are a zoomed-in view of the lowest abundance taxa. Analysis carried out using mothur (v.1.44.3) [49] and classified using the SILVA database (v.138) [50].

At the phylum level, changes in OTU relative abundance pointed to widespread reconfiguration of microbial community structure. Four phyla dominated across all treatments: Proteobacteria, Actinobacteriota, Patescibacteria, and Firmicutes (Figure 3). Notably, fungicide treatments led to an increase in the dominance of these abundant taxa, suggesting selective pressures against rarer members. Systemic fungicides such as Cyprodinil and Difenoconazole elicited dramatic community shifts, including a relative increase in Patescibacteria and reduction in Proteobacteria. Conversely, Azoxystrobin increased Proteobacteria at the expense of Actinobacteriota and Patescibacteria. Rare phyla consistently declined in treated plants, aligning with the observed decrease in diversity (Figure 3).

### The phyllosphere microbiome of the salicylic acid-mediated hyperimmunity mutant, *fhy3 far1*, mitigates non-target fungicide effects

Our earlier research demonstrated that SA-mediated hyperimmunity alters the phyllosphere microbiome by enriching taxa capable of degrading xenobiotics and tolerating oxidative stress. To test if one such microbial community previously examined could confer resilience to pesticide-induced disturbance, the same panel of fungicides was applied to the *fhy3 far1* double mutant, known for its SA-mediated hyperimmune response and spontaneous lesion phenotype.

A total of 1 311 791 high-quality 16S rRNA sequences were obtained from fungicide-treated *fhy3 far1* plants (Supplementary Table 1). NMDS again showed reproducibility but, for *fhy3 far1*, revealed much less distinct separation between treated and control samples (Figure 1). However, alpha diversity analysis revealed that, while fungicide application did reduce microbial diversity in *fhy3 far1* plants, the magnitude of reduction was considerably less than that seen in WT. Prochloraz treatment did not significantly affect diversity, and Cyprodinil actually increased it slightly (Figure 2).

As with WT plants, the dominant phyla in the *fhy3 far1* phyllosphere were Proteobacteria, Actinobacteriota, Patescibacteria, and Firmicutes (Figure 3). However, their relative abundances changed less dramatically upon fungicide application. For instance, Azoxystrobin increased Proteobacteria in *fhy3 far1* similar to WT, but other fungicides did not cause significant shifts. Notably, while Cyprodinil and Difenoconazole increased Patescibacteria, this came at the cost of Actinobacteriota instead of Proteobacteria. Moreover, the reduction in rare taxa seen in WT was absent or reversed in *fhy3 far1*, highlighting the microbiome’s enhanced resilience.

**Figure 4:**
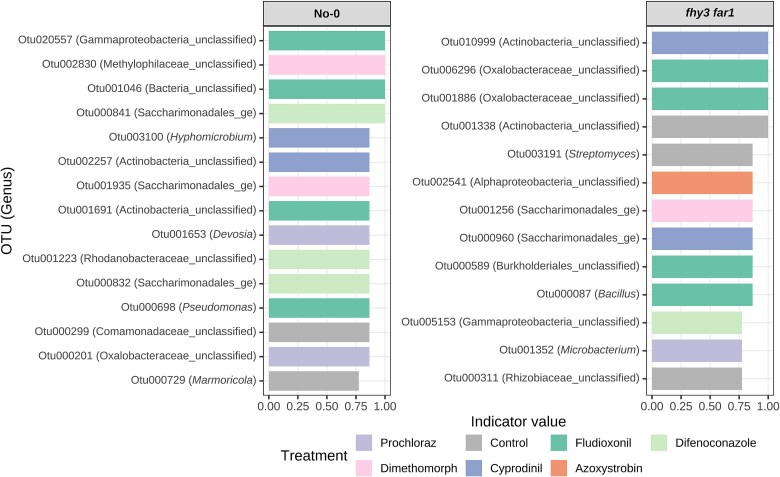
Indicator species analysis of fungicide-treated leaf bacterial communities characterized by classification of 16S rRNA sequences, where indicator OTUs for each treatment were identified separately within each genotype using the IndVal.g statistic with permutation testing (999 permutations) in the indicspecies R package [52].

To examine changes in bacterial composition in more detail at a genus level, we also caried out an indicator species analysis comparing the WT and *fhy3 far1* microbiomes to looks for statistically-significant changes in specific taxa. For both WT and *fhy3 far1* microbiomes, an indicator species analysis comparing control or fungicide-treated plants with the same genotype, revealed a number of bacterial OTUs whose abundance was significantly greater on either control plants or plants treated with a specific fungicide (Figure 4). Indicator taxa were identified for most if not all treatments on both genotypes but were all distinct between WT and *fhy3 far1*. In the WT microbiome, these included, e.g. Otu003100, *Hyphomicrobium*, which was enriched on cyprodinil-treated plants; Otu001653, *Devosia*, on Prochloraz-treated; Otu000698, and *Pseudomonas*, on Fludioxonil-treated plants. In the *fhy3 far1* microbiome, these included, e.g. Otu001352 us, *Microbacterium*, enriched on Prochloraz-treated plants; Otu000087, *Bacillus*, on Fludioxonil-treated; and Otu003191, *Streptomyces*, enriched on control plants (Figure 4). The distinct nature of these indicator taxa between the two plant genotypes also suggests differing impacts of the various fungicides on the microbiomes of WT and *fhy3 far1* plants.

The impact of the fungicide treatments at the genus level was further analysed via a set of absence plots to identify specific OTUs which were eliminated from the phyllosphere microbiome as a result of fungicide treatment (Supplementary Fig. 1). This showed, e.g. that Otu000515, *Nocardioides*, was eliminated by Fludioxonil, Azoxystrobin, Difenoconazole, and Dimethomorph treatment on WT plants in all replicates but was less impacted by Prochloraz and Cyprodinil. Conversely, Otu000345, *Methylotenera*, was only completely removed from all WT replicates by Dimethomorph. These plots also supported the observation that fungicide treatment particularly impacted rare taxa in the WT microbiome, with all of the OTUs lost being rare (<1% relative abundance). The absence plots, again, emphasized the difference in fungicide impact between WT and *fhy3 far1* plants in that the two lists of taxa eliminated by one or more fungicide treatments were quite distinct (Supplementary Fig. 1).

In summary, the distinct responses of WT and *fhy3 far1* mutant microbiota to fungicide application indicate that *fhy3 far1* plants with SA-induced hyperimmunity resisted several of the fungicide-induced disruptions observed in WT, likely due to prior enrichment of xenobiotic-resistant and stress-tolerant microbes. This supports the concept that plant immune signalling can shape a more robust microbiome.

### Fungicide effects are equally significant in total and endophytic microbial communities

To further probe the impacts of fungicides, we evaluated changes within the leaf endosphere microbiome, which resides within plant tissues and is often considered crucial for promoting host health. Systemic fungicides, which are absorbed into the plant, might affect endophytes differently than contact fungicides. Additionally, endophytic communities are closely influenced by host immune responses, especially those involving SA.

WT and *fhy3 far1* plants were treated with Fludioxonil (contact), Azoxystrobin (systemic), and Cyprodinil (locally systemic). Leaf surfaces were rinsed to remove epiphytic microbes, and 16S rDNA from internal tissues was sequenced. Three biological replicates per treatment yielded 3 825 635 high-quality 16S rRNA reads (Supplementary Table 2), which were clustered into OTUs.

**Figure 5:**
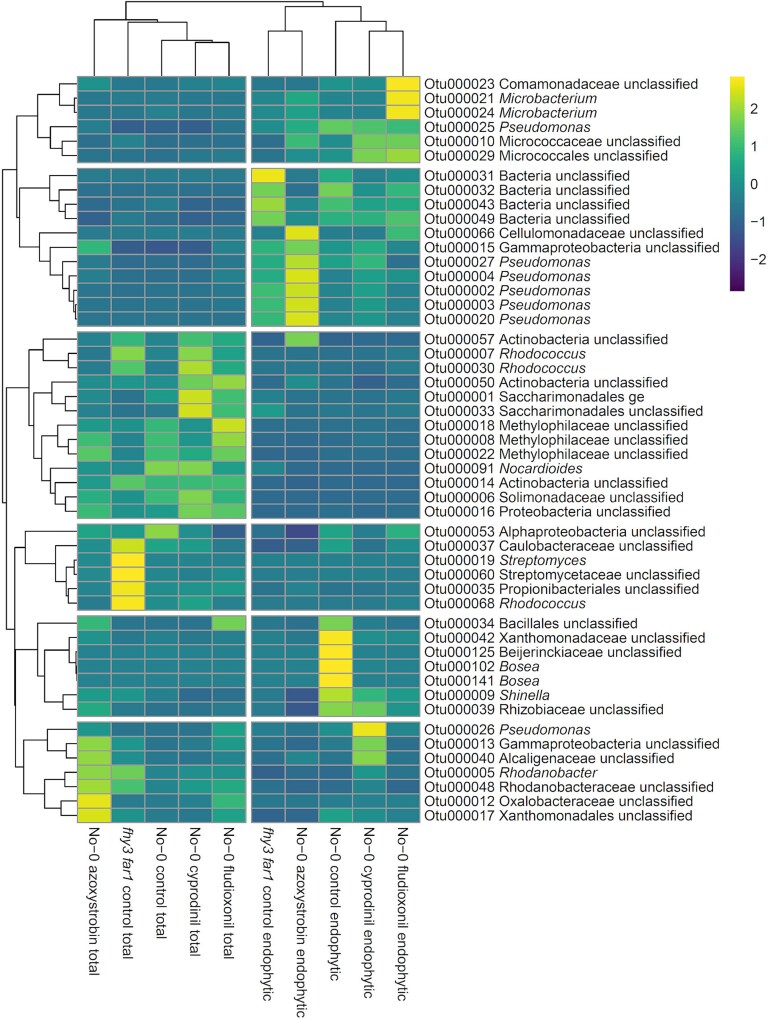
Hierarchical clustering of the top 50 most abundant OTUs from total and endophytic bacterial leaf communities. Clustering was performed using k-means clustering of Euclidean distances of OTU abundances scaled by row to produce Z-scores. OTU abundances were calculated and subsampled down to the size of the sample with the smallest number of reads using mothur (v.1.44.3) [49] and classified using the SILVA database (v.138) [50]. Clustering analysis was performed using the pheatmap package in R.

Hierarchical clustering of the 50 most abundant OTUs revealed clear distinctions between endophytic and total microbiomes across treatments and genotypes, identifying different base communities in interior and exterior leaf tissues (Figure 5). Endophyte communities were enriched in *Pseudomonas* species and unclassified bacteria, while total communities contained higher levels of Actinobacteria, Methylophilaceae, *Rhodococcus*, and Saccharimonadales. Notably, Azoxystrobin-treated samples were the most divergent from controls for both endophytic and total communities. Interestingly, *fhy3 far1* endophyte samples also clustered separately from the majority of other WT samples for both endophytic and total communities, regardless of treatment. Despite their functional differences, endophytic and total microbiota showed comparable clustering in response to fungicide treatment, indicating that fungicides exert broad off-target effects across microbial habitats.

### Host genotype influences endophyte community response to fungicides

**Figure 6:**
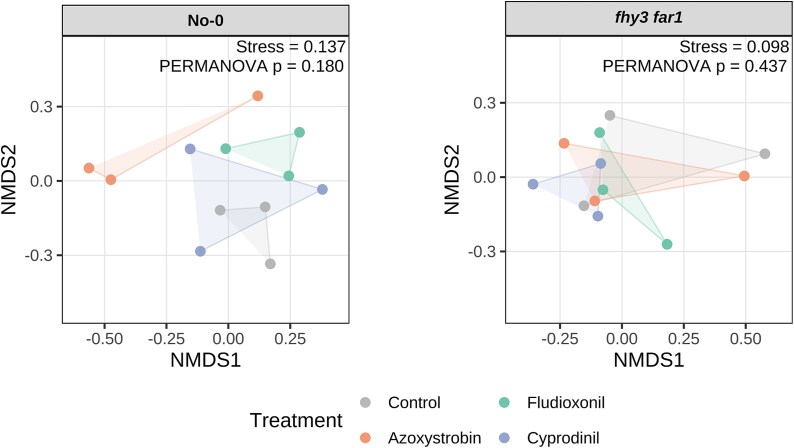
NMDS ordination of fungicide-treated leaf endophytic communities characterized by classification of 16S rRNA sequences. WT and *fhy3 far1 A. thaliana* genotypes, which had been sprayed with fungicide or control at 14 and 28 DAS, were harvested 35 DAS (*n* = 3). All samples were subsampled down to the size of the sample with the lowest number of sequences prior to analysis. Bray–Curtis dissimilarities were calculated from Hellinger-transformed OTU tables (square-root of per-sample relative abundances) prior to NMDS ordination in R [53, 54]. Differences in community composition between treatments within each genotype were assessed using permutational multivariate analysis of variance (PERMANOVA; adonis2, vegan) on the same Bray–Curtis dissimilarity matrix, with significance assessed by permutation. PERMANOVA *P*-values are reported on the ordination panels [55, 56].

To explore genotype-specific responses, NMDS was used to examine community dissimilarities among treatments (Figure 6). Again, NMDS analysis revealed a good clustering of each set of replicates, suggesting good reproducibility of the findings. In WT, Fludioxonil and Azoxystrobin fungicide treatments caused clear divergence from the control group and from each other; though differences were not significant based on PERMANOVA analysis, whereas in *fhy3 far1*, as with the total phyllosphere microbiome, fungicide-treated endospheric communities all showed high similarity to untreated controls, suggesting limited disruption.

### Azoxystrobin diminishes WT endophytic diversity but spares *fhy3 far1* hyperimmune plants

**Figure 7:**
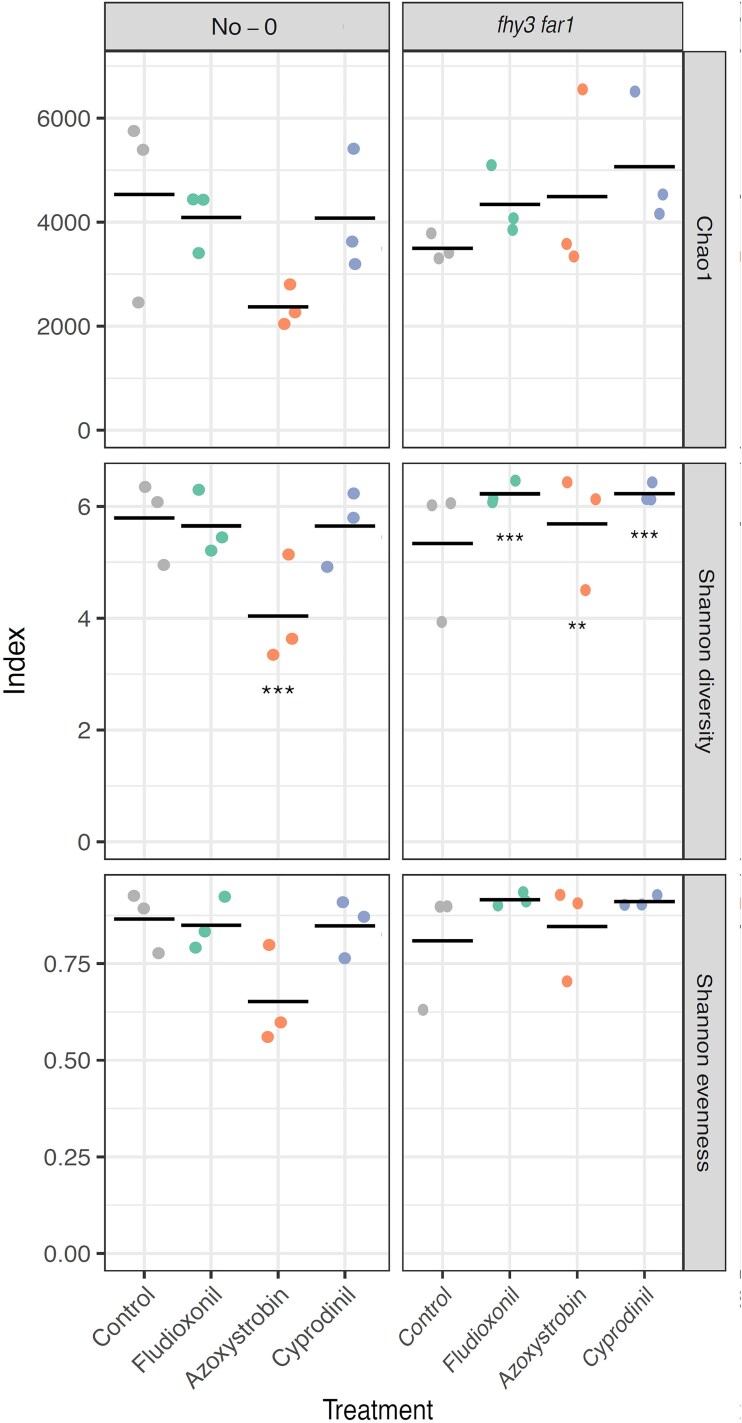
Alpha diversity estimations of the diversity of the bacterial leaf endophytic communities. WT and *fhy3 far1 A. thaliana* genotypes were harvested 35 DAS (*n* = 3). The Chao1 estimator for species richness and the Shannon diversity and evenness indices show the species richness, community diversity and community evenness, respectively. All samples were subsampled down to the size of the sample with the lowest number of sequences before calculating. Alpha diversity metrics were calculated using mothur (v.1.44.3) [49] and inter-condition comparisons were made using the Past4 Diversity t-test function [51]. *P*-values were adjusted using the Benjamini–Hochberg procedure in R. Asterisks represent statistical significance, where “*” indicates *P* < .05; “**” indicates *P* < .01; and “***” indicates *P* < .001.

Species richness, diversity, and evenness were calculated to understand potential impacts on host health (Figure 7). In WT, Azoxystrobin significantly reduced diversity (*P* < .001), while Fludioxonil and Cyprodinil showed no significant impact. Similarly, only Azoxystrobin triggered any clear impact on richness and evenness, causing large decreases in both. In contrast, all treatments slightly increased these metrics in *fhy3 far1*, with statistically significant rises in diversity observed for Fludioxonil, Azoxystrobin, and Cyprodinil (*P* < .001, *P* < .01, and *P* < .001, respectively). Thus, as with the total phyllosphere bacterial microbiome, the endospheric phyllosphere bacterial microbiome of the SA hyperimmune *fhy3 far1* mutant showed a resilience to the impact of fungicide.

### Fungicide-driven compositional shifts in endophytes are altered in the salicylic acid hyperimmune mutant, *fhy3 far1*

**Figure 8:**
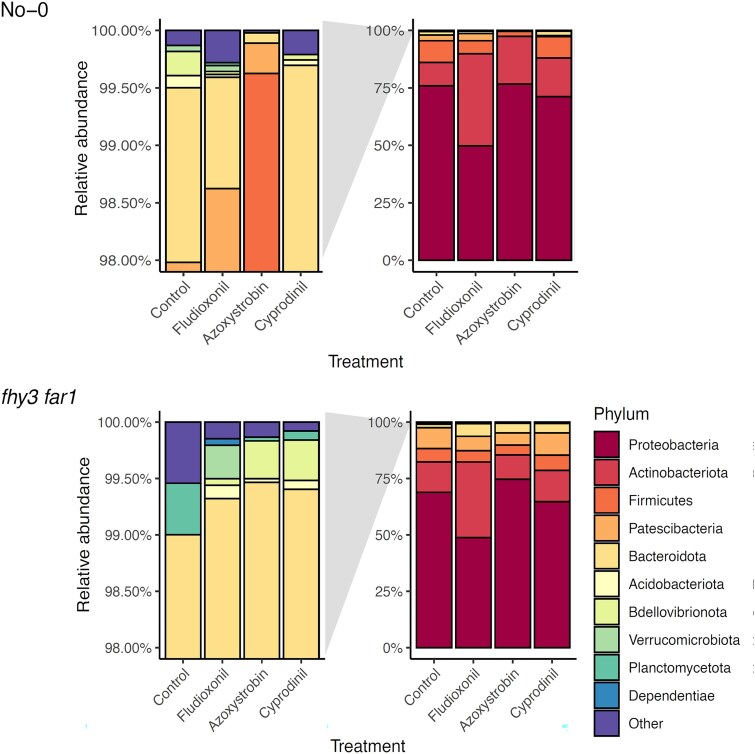
Relative abundances of classified, bacterial leaf endophytic phyla. WT and *fhy3 far1 A. thaliana* genotypes were harvested 35 DAS (*n* = 3). The panels on the left display a zoomed-in view of the least abundant taxa. Analysis carried out using mothur (v.1.44.3) [49] and classified using the SILVA database (v.138) [50].

To further investigate fungicide effects on microbial composition, OTUs were taxonomically classified. Control WT endospheres were dominated by Proteobacteria, followed by Actinobacteria, Firmicutes, and a smaller proportion of Patescibacteria (Figure 8). Compared to the total microbiome, Proteobacteria were more dominant in the endosphere.

Fungicide treatments caused marked shifts in these communities. Fludioxonil decreased Proteobacteria by ~25% while increasing Actinobacteria. Azoxystrobin had minimal effect on Proteobacteria but substantially reduced Firmicutes, a sign of microbiome dysbiosis [11]. Rare taxa were also negatively affected by Azoxystrobin.

In *fhy3 far1*, although Proteobacteria and Actinobacteria displayed similar patterns as in WT, the impact on Firmicutes and rare taxa was significantly buffered. Fludioxonil reduced Proteobacteria similarly in both genotypes, but Azoxystrobin had minimal impact on Firmicutes or rare taxa in *fhy3 far1* (Figure 8), reinforcing our observation that loss of FHY3 and FAR1 confers stability to microbial communities under the chemical stress associated with fungicide treatment.

**Figure 9:**
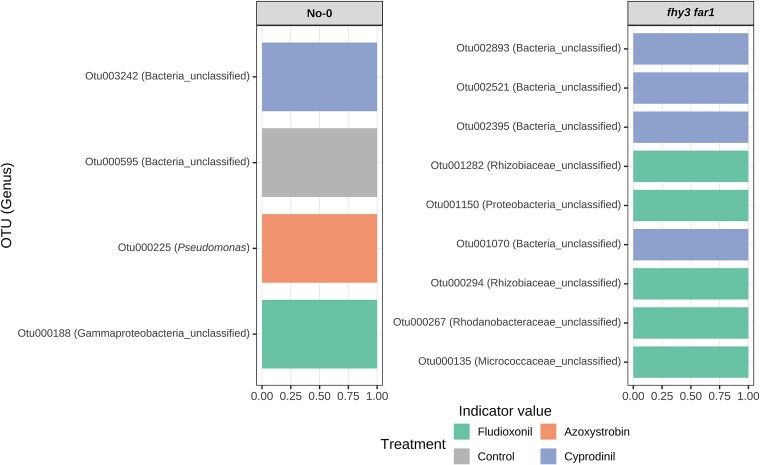
Indicator species analysis of fungicide-treated leaf endophytic communities characterized by classification of 16S rRNA sequences, where indicator OTUs for each treatment were identified separately within each genotype using the IndVal.g statistic with permutation testing (999 permutations) in the indicspecies R package [52].

Again, indicator species analysis revealed a number of specific bacterial OTUs whose abundance was significantly enhanced in response to either control or a specific fungicide treatment in the WT or *fhy3 far1* endospheres (Figure 9). It was notable, here, that indicator taxa were fewer in number among the endosphere population than the total phyllosphere population. Once again, however, these indicator taxa were all distinct between WT and *fhy3 far1*, indicating differing impacts of the fungicide on the two different plant genotypes. Indicator OTUs were identified for control, Fludioxonil, Azoxystrobin, and Cyprodinil-treated WT plants. For example, Otu000225 in the genus, *Pseudomonas*, was identified as enriched within the endospheric phyllosphere of WT plants treated with Azoxystrobin. By contrast, indicator taxa were only identified for Fludioxonil and Cyprodinil-treated *fhy3 far1* plants, consistent with observations that Azoxystrobin had greatly reduced impact on in *fhy3 far1*. None of the specific OTUs identified as indicator taxa in *fhy3 far1* plants were classified to genus level but the indicator taxa for Fludioxonil treatment included three classified to the family level—Otu000294 in the family Rhizobiaceae, Otu000267 in the family Rodanobacteraceae, and Otu000135 in the family Micrococcaceae (Figure 9).

As with the total microbiome, we generated absence plots to identify specific endospheric OTUs eliminated by the fungicides (Supplementary Fig. 2). Fewer endospheric OTUs were eliminated by fungicide treatment but, again, the pronounced impact of Azoxystrobin on endophytic bacteria in wild type seedlings was apparent here, with the vast majority of those taxa negatively impacted showing elimination only in Azoxystrobin treated plants. Generally, once again, the OTUs lost from the WT microbiome were rare taxa (relative abundance <1%); though, two of the taxa eliminated by Azoxystrobin were of higher abundance: Otu000102, *Bosea*, (relative abundance 4.2%) and Otu000125, Beijerinckiaceae unclassified (relative abundance 3%), further emphasizing the marked impact of Azoxystrobin on the WT endospheric microbiome. By contrast, the impact of the three fungicides on the *fhy3 far1* endospheric bacterial microbiome is more even and no higher abundance taxa were eliminated. However, it is noticeable that a high proportion of the OTUs depleted by these fungicides in the *fhy3 far1* endospheric bacterial microbiome is in the genus *Pseudomonas* (Supplementary Fig. 2), implying that the observed buffering conferred by the *fhy3 far1* constitutive immune mutation against the impact of fungicide treatment does not extend to this genus.

Overall, however, the greater stability of the *fhy3 far1* endosphere under fungicide stress reinforces our proposal that host-driven hyperimmunity acts as a protective buffer for the microbiome by enriching the endosphere with xenobiotic-resistant and stress-tolerant taxa.

## Discussion

Our baseline studies explored the impact of synthetic chemical fungicides on the total and endospheric phyllospheric microbial community. While prior research has acknowledged off-target effects of fungicides, this study, to our knowledge, is the most comprehensive in demonstrating such effects on phyllospheric bacteria across multiple fungicide classes [25, 26, 36]. Our results reveal a wide extent of non-target effects caused by these treatments on both microbial groups. In WT *A. thaliana*, all six fungicides tested—each representing a distinct chemical class—consistently reduced bacterial diversity across the total phyllosphere microbiome.

Fungicide treatment also markedly altered community composition with an impact particularly on rare taxa, suggesting that these depleted taxa have particular sensitivity to the applied synthetic compounds. However, indicator species analysis also revealed some enriched, rare OTUs, in the phyllosphere of plants treated with fungicide. It has been demonstrated previously that presence of environmental pollutants in the phyllosphere favors bacterial strains with pollutant-degrading capabilities [57], which implies that such strains selected on fungicide-treated plants may be those which have the ability to degrade the fungicide compounds involved. Consistent with this, many of these indicator taxa represented genera previously associated with degradation of pollutants. H*yphomicrobium*, enriched on cyprodinil-treated WT plants, is a genus of methylotrophic bacteria commonly found in the plant leaf surface, utilizing carbon sources like methanol and methane but also potentially degrading harmful compounds like chloromethane [58]. The genus, *Devosia*, enriched on Prochloraz-treated WT plants, is known for its metabolic versatility, including the potential for biodegradation of aromatic compounds [59], while *Pseudomonas*, enriched on Fludioxonil-treated WT plants are major, diverse inhabitants of the phyllosphere that can act as active agents in bioremediation [60, 61].

Consistently, the endophytic bacterial communities showed more resilience to these treatments. Only a subset of fungicides—specifically, the systemic ones—were tested further for their impact on the endospheric microbiome. Fludioxonil was chosen over Prochloraz among contact fungicides due to broader regulatory approval, while Azoxystrobin and Cyprodinil represented systemic fungicides with different mechanisms of action. Cyprodinil works by inhibiting the biosynthesis of methionine, and by disrupting the secretion of hydrolytic enzymes from fungal cells [62], whereas Azoxystrobin inhibits fungal respiration by blocking mitochondrial electron transport [63]. It is important to note, however, that Azoxystrobin is also known to induce significant physiological changes in treated plants, referred to as the “green effect,” which includes delayed senescence, increased chlorophyll content, and enhanced stress tolerance [64]. The fungicide increases the activity of antioxidative enzymes in treated plants which helps reduce reactive oxygen species, protecting plants from oxidative damage [65]. Notably, only Azoxystrobin significantly reduced bacterial diversity in the endophytic communities of WT plants. The minimal impact of other fungicides on these endophytes may reflect their deep integration with host physiology, suggesting stronger protective mechanisms or selective advantages for resilient taxa. There are also, of course significant differences in action between systemic and contact fungicides. For example, by altering cuticle permeability, nutrient leakage, or specialized metabolite profiles that mediate microbial colonization and stress tolerance [66].

However, the observed effect of Azoxystrobin is particularly concerning. This fungicide, which inhibits mitochondrial cytochrome electron transport, is known to suppress bacteria unable to metabolize it [40, 67]. Its application significantly reduced the presence of Firmicutes—a group linked to plant health—indicating a possible shift toward microbial dysbiosis [11]. Indeed, Firmicutes have proved to be able to promote plant growth through a range of direct mechanisms. They can enhance the uptake of essential nutrients, such as phosphorus, nitrogen, and iron (siderophore production), making them more accessible to plant roots [68]. Many species produce plant growth-regulating compounds, including indole-3-acetic acid (IAA), which boosts root system development [68]. In addition, they can help plants withstand abiotic stresses like drought, salinity, and high temperatures by regulating hormone levels, such as reducing ethylene through ACC deaminase production [68]. Firmicutes can also act as biological control agents (biopesticides) due to their production of antimicrobial that inhibit the growth of pathogens [69]. At the same time, they can also trigger Induced Systemic Resistance, priming the plant’s own defence mechanisms [69].

Both epiphytic and endophytic bacteria contribute to plant health [70]. However, because of their location within plant tissues, in intercellular spaces and within vascular tissue, endophytes are more directly able to influence plants via production of diffusible compounds [68]. For example, in a study of epiphytes versus endophytes in grapevine, the ability of bacteria to suppress the growth of pathogens through production diffusible metabolites was far more widespread among endophytes than among epiphytes [71]. Thus, disruption of endophytic bacterial communities could have more severe consequences than disturbances in surface microbiota. Given the widespread use of strobilurin-class fungicides like Azoxystrobin, it is critical to investigate whether similar negative effects occur with other members of this group [72].

The *fhy3 far1* hyperimmune mutant, characterized by constitutive SA signalling, displayed notable resistance to many fungicide-induced microbiome changes. In this mutant, community composition in the phyllosphere remained more stable, and bacterial diversity either remained unchanged or increased—patterns typically associated with healthy plant–microbe interactions [11].

Within the endophytic compartment, only Fludioxonil affected community structure by enriching Firmicutes, while other treatments showed little to no impact.

We hypothesize that *fhy3 far1*’s altered microbiome composition, previously shown to be enriched in xenobiotic degradation traits in common with another elevated SA signalling mutant [21], may grant increased resilience to abiotic chemical stressors. The hypersensitive response phenotype of this mutant may have already selected for robust microbial partners, which in turn are more resistant to disturbance. Additionally, the plant immune system itself may be responsible for mediating microbial homeostasis, particularly within the endosphere, where immune regulation is tightly coupled to community structure [13, 73]. SA is a known regulator of systemic acquired resistance, a pathway that primes the plant for broad-spectrum defence and may incidentally shape the microbiome toward more stress-resilient communities [74–76].

Based on our findings, several important conclusions emerge. Firstly, multiple synthetic fungicides exert substantial non-target effects on the phyllospheric microbiome, reducing both community diversity and compositional stability. These impacts were especially severe in the total phyllosphere but, in some cases, extended to the endophytic compartment as well. Azoxystrobin, in particular, was shown to significantly disrupt the endosphere, suggesting that not all fungicides are equal in their off-target effects.

Secondly, SA-mediated immunity in *fhy3 far1* mitigated many of the negative impacts of fungicide application, highlighting the potential of plant immune status to buffer against external chemical disturbances. This opens up intriguing possibilities for incorporating plant immune modulation into IPM strategies. If certain microbiota traits selected by SA signalling also confer resilience to fungicide exposure, then such communities could serve as ideal candidates for microbiome engineering or as components of bioinoculants.

Thirdly, the findings suggest that it may ultimately be possible to identify specific bacterial taxa that remain stable in the presence of fungicides. Such a development would, of course, require functional validation but would have clear agricultural implications. These resilient microorganisms could be developed into biostimulants capable of sustaining plant health even under chemical stress. Future studies employing full metagenomics and whole genome sequencing of phyllosphere inhabitants would help identify the genetic basis of this resilience, providing crucial insight for developing targeted, low-impact crop protection strategies.

Ultimately, our results suggest that strategic manipulation of the microbiome—either through selection of resilient taxa or by leveraging SA-mediated immunity—may reduce the collateral damage of fungicide application. Combining these biological approaches with reduced chemical inputs could form the basis of a sustainable, hybrid IPM model. Such an approach would not only maintain crop productivity but also minimize ecological disruption and slow the evolution of resistance in plant pathogens.

## Supplementary Material

Supplementary_Material_ycag102

## Data Availability

The data underlying this article are available in the NCBI SRA repository at https://www.ncbi.nlm.nih.gov/sra, and can be accessed with BioProject ID: PRJNA1307028.
