# Supplementary material for: Fungicides disrupt total and endophytic phyllosphere bacterial communities but a salicylic acid hyperimmune mutant shows microbiome resilience
Source: ISME Commun. 2026 Apr 14;6(1):ycag102. doi: 10.1093/ismeco/ycag102 (PMC13174326; doi:10.1093/ismeco/ycag102)
Supplement: Supplementary_Material_ycag102 [file supplementary_material_ycag102.docx]

**Fungicides disrupt total and endophytic phyllosphere bacterial communities but a salicylic acid hyperimmune mutant shows microbiome resilience**

Stacey A. Vincent and Paul F. Devlin

Supplementary Material

Supplementary Tables

Supplementary Table 1 Number of high-quality, classified 16S rRNA sequences produced by NGS across WT and fhy3 far1 samples

| Genotype | Treatment | No. of 16S rRNA sequences | | |
| --- | --- | --- | --- | --- |
|  |  | Replicate 1 | Replicate 2 | Replicate 3 |
| WT | Control | 31,311 | 59,276 | 97,196 |
|  | Prochloraz | 199,583 | 88,449 | 55,212 |
|  | Azoxystrobin | 58,384 | 2,497 | 13,955 |
|  | Fludioxonil | 107,479 | 39,013 | 57,182 |
|  | Cyprodinil | 123,849 | 65,248 | 50,612 |
|  | Difenoconazole | 82,060 | 50,057 | 84,108 |
|  | Dimethomorph | 151,472 | 408,179 | 119,593 |

| Genotype | Treatment | No. of 16S rRNA sequences | | |
| --- | --- | --- | --- | --- |
|  |  | Replicate 1 | Replicate 2 | Replicate 3 |
| *fhy3 far1* | Control | 13,297 | 39,955 | 88,784 |
|  | Prochloraz | 57,553 | 63,990 | 121,864 |
|  | Azoxystrobin | 52,397 | 4,196 | 30,887 |
|  | Fludioxonil | 59,554 | 30,171 | 40,137 |
|  | Cyprodinil | 112,928 | 34,026 | 36,900 |
|  | Difenoconazole | 88,165 | 97,055 | 30,104 |
|  | Dimethomorph | 104,459 | 81,897 | 123,472 |

Supplementary Table 2. Number of high-quality, classified 16S rRNA sequences produced by NGS across leaf endophytic A. thaliana WT and fhy3 far1 samples

| Genotype | Treatment | No. of 16S rRNA sequences | | |
| --- | --- | --- | --- | --- |
|  |  | Replicate 1 | Replicate 2 | Replicate 3 |
| WT | Control | 74,077 | 128,674 | 614,436 |
|  | Fludioxonil | 46,974 | 280,487 | 87,932 |
|  | Azoxystrobin | 106,078 | 261,495 | 188,724 |
|  | Cyprodinil | 85,436 | 314,619 | 284,864 |
| *fhy3 far1* | Control | 18,773 | 159,937 | 76,875 |
|  | Fludioxonil | 22,090 | 197,120 | 109,240 |
|  | Azoxystrobin | 62,220 | 191,263 | 33,865 |
|  | Cyprodinil | 56,828 | 312,721 | 110,907 |

***
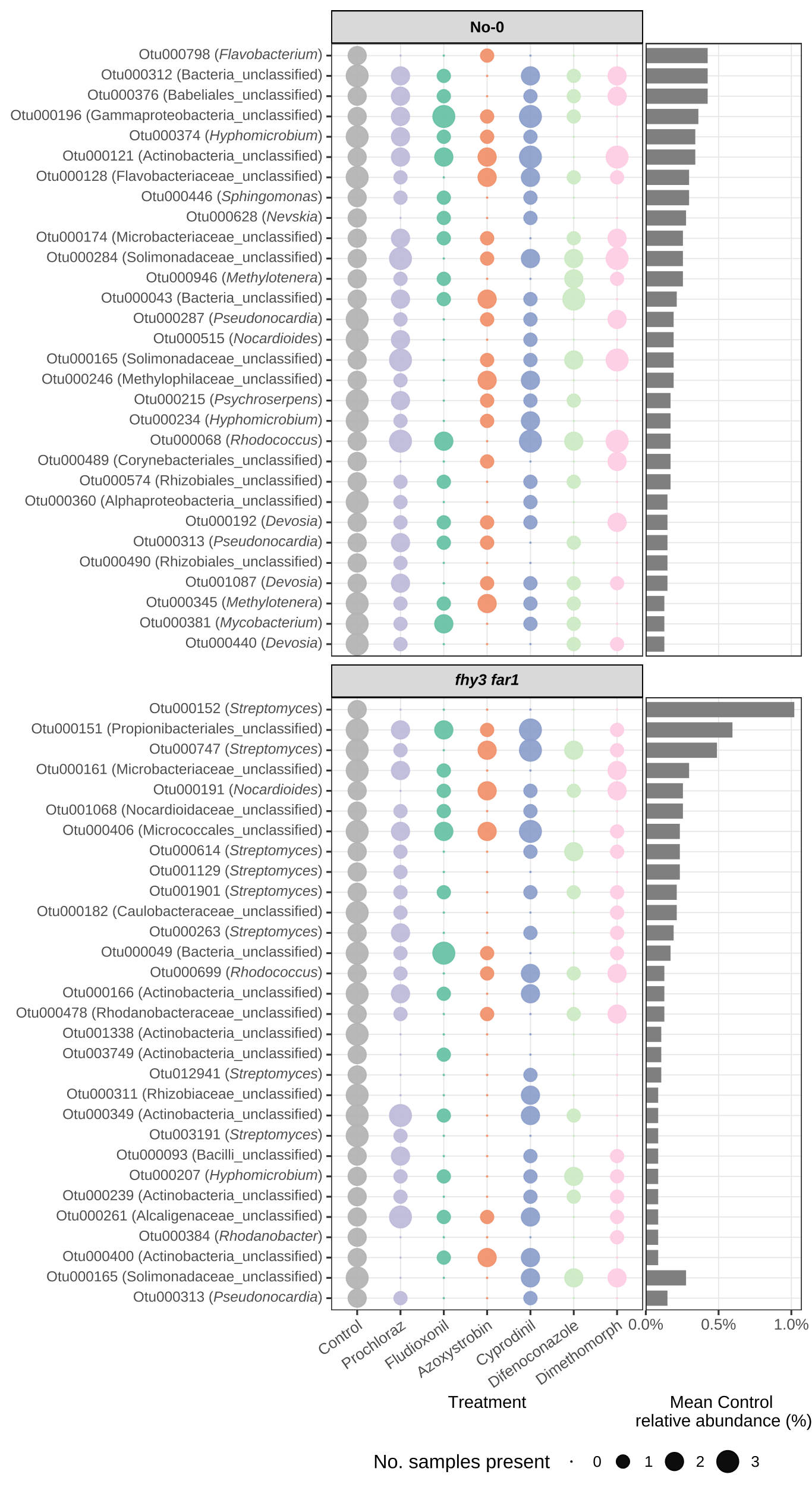
***Supplementary Figures

***Supplementary Figure 1. Absence/prevalence plots of fungicide-treated leaf bacterial communities highlighting OTUs detected in Control but absent under ≥1 fungicide treatment.*** *For each genotype (No-0 and* fhy3 far1*), OTUs were selected if they were detected in at least two Control replicates, had a minimum total Control read count of 2, and were completely absent in at least one fungicide treatment. Points are coloured by treatment, and point size indicates the number of replicates in which the OTU was detected for that treatment (0–3). Bar plots on the right show the mean relative abundance of each OTU across Control samples for the corresponding genotype.*

***
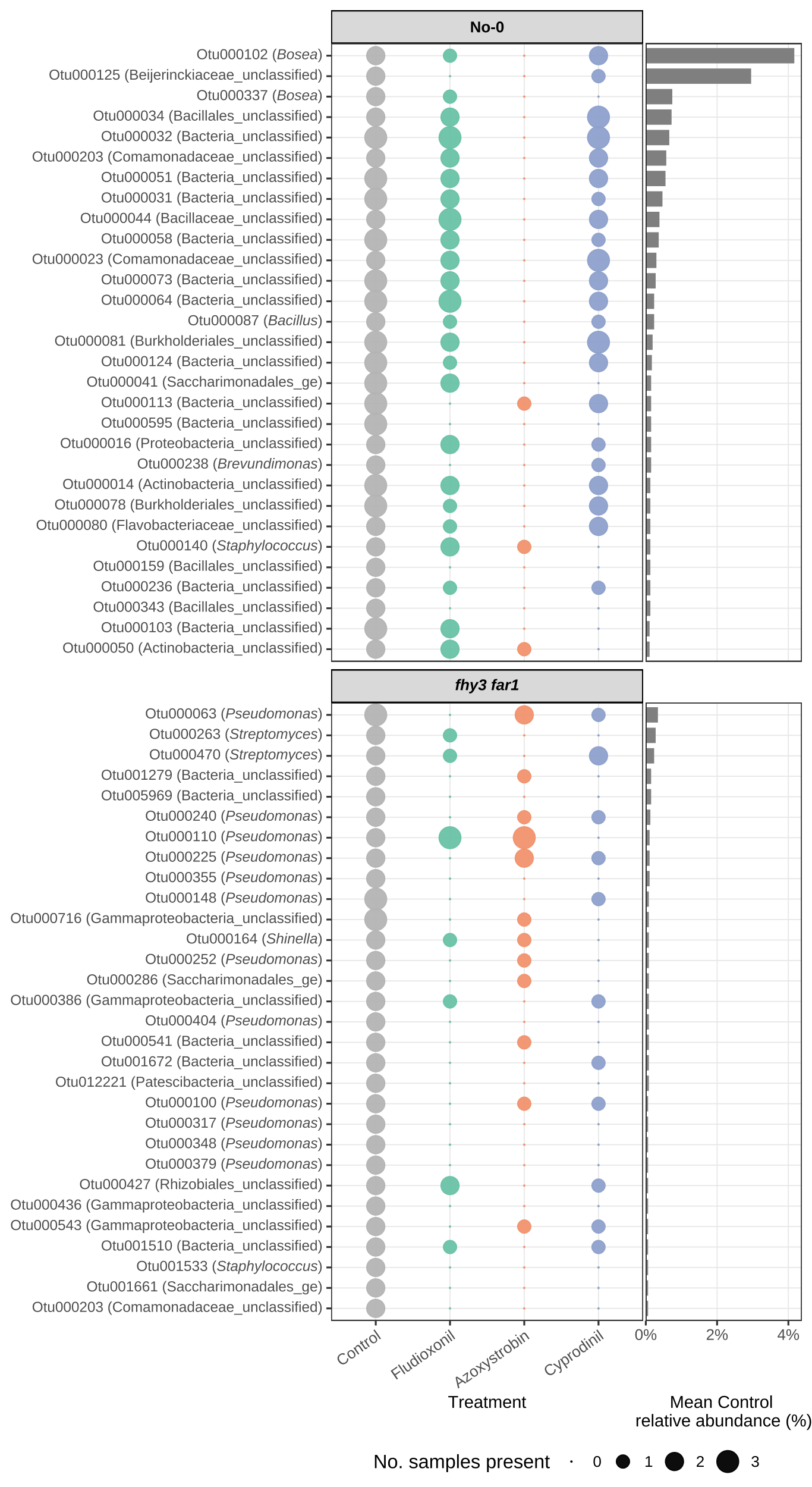
***

***Supplementary Figure 2. Absence/prevalence plots of fungicide-treated leaf endophytic communities highlighting OTUs detected in Control but absent under ≥1 fungicide treatment.*** *For each genotype (No-0 and* fhy3 far1*), OTUs were selected if they were detected in at least two Control replicates, had a minimum total Control read count of 2, and were completely absent in at least one fungicide treatment. Points are coloured by treatment, and point size indicates the number of replicates in which the OTU was detected for that treatment (0–3). Bar plots on the right show the mean relative abundance of each OTU across Control samples for the corresponding genotype.*
